# A large maxillary cemento-ossifying fibroma superimposed with solitary bone cyst documented over 18 years: A case report

**DOI:** 10.1016/j.ijscr.2020.03.011

**Published:** 2020-03-07

**Authors:** Sarmad Aburas, Patrick Bandura, Ali Al- Ibraheem, Sebastian Berger, Marius Meier, Dritan Turhani

**Affiliations:** Centre for Oral and Maxillofacial Surgery, University of Dental Medicine and Oral Health, Danube Private University, Steiner Landstraße 124, 3500 Krems-Stein, Austria

**Keywords:** CCOF, central cemento-ossifying fibroma, CF, cementifying fibroma, COF, central odontogenic fibroma, CT, computed tomography, DVT, digital volume tomography, FD, fibrous dysplasia, JOF, juvenile ossifying fibroma, OF, ossifying fibroma, PCOF, peripheral cemento-ossifying fibroma, SBC, solitary bone cyst, WHO, World Health Organisation, Case report, Cemento-ossifying fibroma, Juvenile ossifying fibroma, Solitary bone cyst, Fibrous dysplasia

## Abstract

**Introduction:**

Cemento-ossifying fibromas are rare, benign lesions of the head and neck regions. The origin of these lesions can be traced to the periodontal ligament, because its cells are able to form cement, lamellar bone, or fibrous tissue.

**Case presentation:**

A rare case report of a young Caucasian female with a COF is described including follow-ups. She remained untreated for several years despite early radiographic findings of the lesion without a definitive diagnosis. The patient ultimately underwent radical surgery and the whole lesion was removed. The subsequent histological examination confirmed the clinical diagnosis of a COF. Since then, the patient has been under regular clinical and radiological follow-ups.

**Discussion:**

This rare case report of a COF, documented over 18 years, shows the importance of consecutive therapy after radiologic and clinical findings, as such lesions continue growing and may subsequently lead to severe medical conditions. Therefore, complete surgical resection of COFs is advised to achieve good results in terms of health and recurrence.

**Conclusion:**

Long-term follow-up of patients is required as recurrences can occur for up to 10 years following treatment. Well-planned radical and wide surgical resection of these lesions has proven not only to be effective in eliminating the aetiological factors, but can also achieve decent bone regeneration and aesthetic results with almost no deformation in the surgical site.

## Highlights

• Cemento ossifying fibromas are benign rare odontogenic lesions.
• The lesion was surgically removed successfully and the patient has been followed up for 18 years, with no sign of recurrence.
• Early diagnosis is key to prevent the large growth of the lesion which often results in multiple tooth loss.
• Close follow up is important due to the possible recurrence.
• Unusual location of a COF in the maxilla, with the superimposition of a solitary bone cyst.

## 1 Introduction

Cemento-ossifying fibroma (COF) is a rare slow benign fibro-osseous tumour arising from the periodontal ligament [1]. It is formed by a layer of fibrous connective tissue that encircles the roots of the teeth. The lesion contains multipotential cells that are capable of forming cementum, lamellar bone, and fibrous tissues [2,3]. Its histological appearance shows numerous clusters of cement embedded in the fibrous tissue with areas of splintered bone fragments [4]

In general, COF is more prevalent in females between 30 and 40 years old and is more common in the mandible (70 %) than in the upper jaw (maxilla posterior region, 22 %) [5]. It is usually discovered via orthopantomogram during regular visits to the dentist. The lesion can present as a defined multilocular mixed radiolucent and radio-opaque mass with marginal sclerosis, which differentiates it from fibrous dysplasia. It can also present as an unilocular radiolucent lesion at an early stage, or more of a radiopaque mass at later stages [6].

Cemento-ossifying fibromas are round or ovoid, slow growing masses that usually affect the adjacent teeth and can cause root resorption [7]. Permanent growth may lead to an enlargement of the jaw, resulting in an aesthetical and functional deformation of the affected jaw [8].

The origin of COF is considered unknown. Wenig et al. suggested that past traumata or local irritants are possible causes for the development of a COF [9].

The term “cemento-ossifying fibroma” was issued by the fourth edition of the WHO classification of 2017, describing it as a benign mesenchymal odontogenic tumour [10].

Two subtypes of COF are reported in the literature based on age (adult or juvenile), histological content (cementoid or osteoid), and histological pattern (psammomatoid or trabecular) [6], psammomatoid juvenile ossifying fibromas and trabecular juvenile ossifying fibromas.

The psammomatous type is primarily found around the paranasal sinuses and orbits. It has small uniform spherical ossicles resembling psammoma bodies. The trabecular type is found mostly in the jaws and is characterised by fibrous trabecula [11].

Juvenile ossifying fibromas (JOF) are further categorised as juvenile active ossifying fibromas and juvenile aggressive ossifying fibromas, which occur in children younger than 15. The appearance of more than three subtypes in a single lesion is not uncommon, for example, within psammomatoid juvenile cemento-ossifying fibromas [12].

According to the site of origin, a COF can be classified as a peripheral cemento-ossifying fibroma (PCOF) or a central cemento-ossifying-fibroma (CCOF).

An early diagnosis and consecutive therapy can prevent complications such as pathological fractures. Complete surgical removal of the lesion remains the gold standard [8]. Regular follow-up is recommended to counter possible recurrences.

The aim of this case report is to describe the slow but permanent growth of a COF and the clinical, radiological, and histological characteristics. To the best of our knowledge, there is no such case in the existing literature of a cemento-ossifying fibroma documented over such a long period of 18 years, including preoperative and postoperative time.

The patient was managed at our academic institution. The present case is reported in accordance with the SCARE criteria [13].

## 2 Case presentation

We report the case of a 19-year-old Caucasian woman diagnosed with a cyst in her upper jaw, expanding from regions 23–26.

**Fig. 1:**
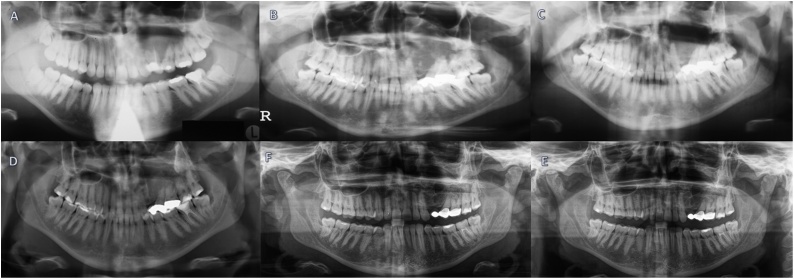
*A,* orthopantomogram revealing a cystic lesion between teeth 23-26. *B,* preoperative orthopantomogram in year 2007. *E,* Postoperative orthopantomogram in year 2007. *C*, Postoperative orthopantomogram showing adequate removal of the lesion in 2010. *D*, orthopantomogram obtained in 2017 showing no signs of recurrence and good bone remodelling. *E*, orthopantomogram obtained in 2019. It is clear to see that the situation hasn’t changed during the years 2017 and 2019.

The first orthopantomogram was conducted in January 2001 by her local dentist during a routine examination. The orthopantomogram revealed a large radiolucent, multilocular periapical lesion expanding from regions 23–26, reaching into the maxillary sinus. The edges of the lesion appeared sclerosed (Fig. 1).

The involved teeth showed no signs of root resorption and all tested positive for vitality. The patient had an unremarkable drug, family, and psychological history and showed no clinical symptoms or pain, which is considered a typical characteristic of COF.

The attending dentist referred the patient to the General Hospital in Vienna (AKH) and a biopsy was performed. During the operation, tooth 24 had to be extracted.

The following analysis of the specimen combined with the clinical and radiological findings led to the diagnosis of a solitary bone cyst.

Unfortunately, the patient refused any further treatment, including a cystectomy.

The patient did not reappear from 2001 until 2007 due to the absence of any clinical symptoms or pain. In late 2007, she was referred to the General Hospital in Vienna, Department of Maxillofacial Surgery.

She was suffering from intraoral swelling on the left side of her maxilla and complained about a strong feeling of dull pressure in the left eye region. During the ensuing 6 years, the already large cyst had expanded even further, pushing apart the roots of the adjacent teeth. Throughout the clinical oral examination, painless swelling of the left maxilla (regions 24–26) was observed. All of the patient’s teeth, including those inside the lesion, reacted positively to the vitality check.

A newly taken orthopantomogram showed the now massive extension of the tumorous lesion, covering almost the entire part of the left maxillary sinus. The lesion had a diameter of approximately 3.4 × 3.2 cm.

**Fig. 2:**
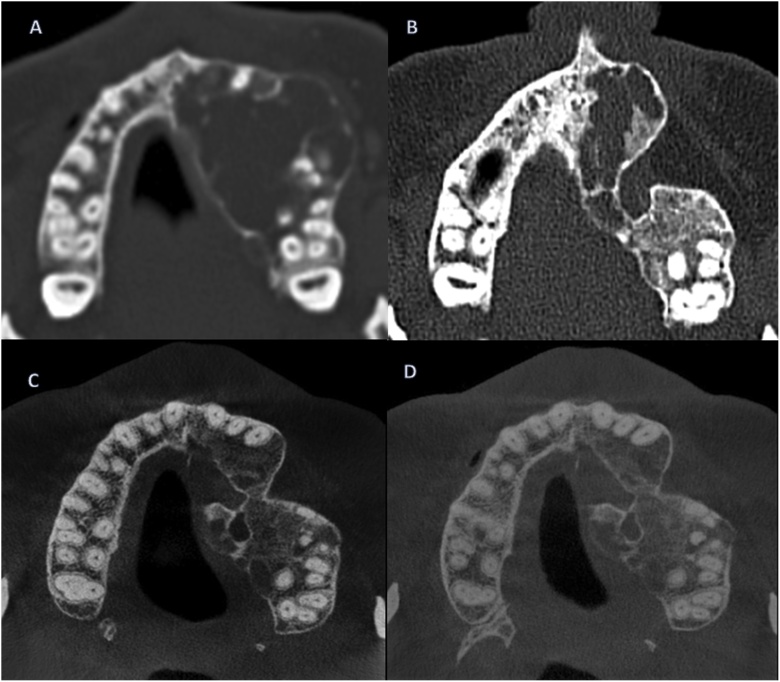
*A*, Preoperative Computer tomography in 2007 showed a large distension within the left sinus maxillaris in size of 3,4 × 3,2 × 2 cm. Signs of sclerosis are clear within the marginal edges of the cystic lesion. *B,* Computer Tomography during a follow up appointment revealed cystic changes in operated region, prompting another operative removal of the recurrent cyst*. C and D,* Computer Tomography taken in 2017 and 2019 showed no signs of recurrence and good bone remodeling.

After informing the patient about the findings and upcoming treatment, she provided consent for the necessary operation. A preoperative computer tomography scan showed a 3.4 × 3.2 × 2 cm distension within the left maxillary sinus (Fig. 2). The marginal edges of the cystic lesion appeared sclerosed. There was no sign of infiltration of the surrounding soft tissue.

**Fig. 3:**
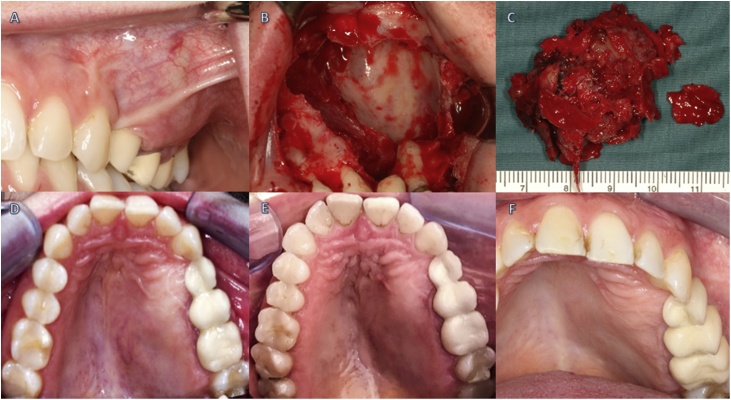
*A,* vestibulo-palatal distention in the 2nd quadrant, 2007. *B*, Cystectomy in year 2007. *C,* Cystectomy in year 2009. *D*, a palatinal view of the lesion in year 2017 during a follow-up appointment. *E*, a palatinal view of the lesion in year 2019 during a follow-up appointment, it is clear to see that the lesion has not changed between year 2017 and year 2019. *F*, frontal view of the lesion in year 2019.

Two weeks later, the patient underwent surgery under general anaesthesia by the head of the Department of Maxillofacial Surgery. The lesion was removed using an intraoral approach, and a mucoperiosteal flap was placed from regions 23–28. After bone windowing, the tumour was removed completely (Fig. 3).

The patient received postoperative antibiotics, a non-steroidal anti-inflammatory agent, and a proton pump inhibitor and was discharged after a few days.

The resected 3.4 × 3.2 × 2 cm specimen was histopathologically examined. Microscopically, the bone section was deformed by a cystic lesion filled with blood, and the cyst wall of the loose connective tissue was covered with granulation tissue of different ages.

**Fig. 4:**
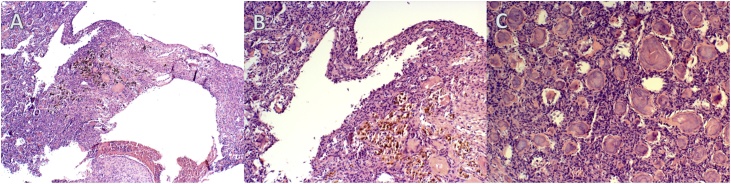
*A,* Histological specimen showing SBC and COF (enlargement 100 μm). *B,* Histological specimen (enlargement 200 μm). *C,* Histological specimen showing COF in detail (enlargement 200 μm).

Each trabecula was lined with distinct and thick osteoblast segments in different places. The osseo-trabecula had sections of dense cellular lesions consisting of small spheres of a cementitious material bounded by plump, fuzzy cells with many vascular gaps in between (Fig. 4).

This morphological description of the lesion matched a solitary bone cyst plus focal sections of cemento-osseous dysplasia. There were no signs of malignancy.

The patient attended regular follow-up appointments. Six months after surgery, she remained symptom-free and was in the ninth week of pregnancy.

During a regular follow-up CT in 2009, new cystic changes were found within the left maxilla from regio 22–24. Another cystectomy was performed under general anaesthesia by the head of the Department of Maxillofacial Surgery at the University Hospital St. Pölten.

Using an intraoral approach, an incision from region 22–26 was preformed and the raised mucoperiosteal flap was carefully extended to expose the incisal nerve. After bone windowing the cystic like lesions were revealed and then removed in several fragments. A fibrin sealant, Tachosil®, was used on the operated tissues to stop the bleeding. This minimally invasive approach enabled the patient’s rapid recovery with no complications.

The patient was given postoperative antibiotics, a non-steroidal anti-inflammatory agent, and a proton pump inhibitor and was discharged in stable condition a few days later.

The removed fragments were histologically examined and showed a calcified material that consisted of various differently sized and generally small spherical basophilic cement elements with cellular fibrous stroma. The final diagnosis was revised to cemento-ossifying fibroma with cystic formations matching a solitary bone cyst.

A radiological follow-up in 2010 showed no signs of recurrence.

The residual sinus walls were clearly sclerosed. In addition, inflammatory residual remnants less than 1 cm were found in the surgical site. The partially lytic, partially sclerosed changes in the alveolar process of the left maxilla remained, with increasing sclerosing at regions 22 and 23. The local status of the teeth remained unchanged.

Since then, the patient has been under regular clinical and radiological follow-up.

DVTs taken in 2016, 2017, and 2019 showed insignificant changes in the left maxilla.

To date, approximately 10 years after the second operation, there have been no signs of recurrence and the patient is clinically symptom-free. Moreover, the patient is satisfied with the outcome of treatment.

## 3 Discussion

In summary, this kind of lesion was first described in 1872 by Menzel as a type of ossifying fibroma in the mandible. Since 1968, cementum informalities containing tumours have been categorised as COFs [3]. The term “COF” is most commonly used to describe fibro-osseous lesions that originate from the periodontal ligament and expand from the cementum into the neighbouring bone [14,15].

According to the 2017 WHO classification, the term “cemento-ossifying fibroma” was categorised as a type of mesenchymal odontogenic tumour [10]. If bone dominates, it is considered an ossifying fibroma; however, if cementum is present, it is considered a cementifying fibroma. The term “cemento-ossifying ﬁbroma” is used if both hard tissues are present [10].

There are two types of ossifying fibromas, central and peripheral. The central type arises from the cells of the periodontal ligament in the apical area, causing the expansion of the lamina dura. The peripheral type occurs in the soft tissues of the teeth-bearing areas [16].

Similarly, solitary bone cysts (SBCs) appear clinically symptomless and are often discovered accidently. The SBC is a benign cavity in the bone that classifies as a pseudocyst without lining epithelium. SBC is either empty or is filled with fluid [17] and is also known as traumatic bone cyst, traumatic bone cavity, simple bone cyst, idiopathic bone cyst, or hemorrhagic bonce cyst [18,19]. They present as swelling without tooth pain, although the neighbouring teeth are most likely cold-positive and their roots may suffer from displacement, resorption, hypersensitivity, or fistula with pathological fractures as common symptoms. The proximal femoral and humeral metaphysis, molars, and premolar regions of the lower and upper jaws are the most common areas for solitary bone cysts to occur [20]. The etiology of the SBC is considered to be unclear. However, there are some theories about the origin of this lesion; such as trauma and developmental-related causes [18,21]. SBC has a higher prevalence to males than to females, with most cases occurring in the second decade of life [22].

A similar disease described in the literature is the aneurysmal Bone Cyst (ABC). In contrast to the SBC, however, the ABC shows a clearly more aggressive clinical behaviour [18]. Surgical exploration followed with curettage of bone walls is considered to be the most suitable method to manage and treat such lesions [17]. Two subtypes of OFs are reported in the medical literature, based on age (adult/juvenile), histological content (cementoid/osteoid), and histological patterns (psammomatoid/trabecular) [6].

Juvenile ossifying fibromas (JOFs) commonly occur aggressively during the beginning of adulthood when patients are undergoing hormonal and biological changes. This applies to our patient, as she was 19 years old when the cyst was first discovered.

In 2002, El-Mofty presented the most relevant classification, identifying 2 categories according to their histological criteria: trabecular juvenile ossifying fibroma (TrJOF) and psammomatoid juvenile ossifying fibroma (PsJOF). The site of occurrence is a clinical feature that can help differentiate the two subtypes. PsJOF primarily occur in the paranasal sinus area, and TrJOF tend to occur more in the maxilla. In general, mandibular and extracranial involvement is rare [11].

The management and prognosis of JOFs are uncertain. In some cases, there are minimal symptoms, and in other cases, especially in very young patients, the tumour has very local behaviour with a high recurrence rate (30–58 %), Therefore, it is recommended that these locally aggressive neoplasms be treated with wide surgical resection rather than conservative curettage [11].

For many years, the origin of COFs was considered the periodontal ligament [23]. However, recent microscopic studies of lesions in the frontal, temporal, sphenoid, and ethmoid bones made this debatable.

The lesion can develop from the periodontal membraneAlso, he mesenchymal cells within the mesodermal germ layer can differentiate and develop into multiple cell types producing a complex tumour [24]. These two conditions combined can perhaps explain the multiple histological findings in our case.

The aetiology of COFs is yet unknown, but trauma may act as a predisposing factor, which suggest a connective-tissue-reactive aetiology rather than a neoplastic aetiology [25]. It has been proposed that traumas or dental extractions may be stimulating factors due to the remaining periodontal membrane cells attached to the wall of the alveolus [26,27].

Clinically, COFs are usually painless, spherical, or ovoid, intra-bony symptomless, slow growing masses. However, in certain cases, pain or paraesthesia may be present if the adjacent nerve is affected.

COFs can also cause sinus obstruction, facial deformity, proptosis, and intracranial complications, although they can remain symptom-free without affecting the vitality of the surrounding teeth or causing any signs of necrosis during early stages of development. In some rare cases, the lesions can grow massively, causing significant cosmetic and functional deformities [28].

In general, the lesions are firm in consistency, subject to the degree of mineralisation within. Intra-orally, the lesions are covered with normal mucosa with no signs of associated adenophathies [24].

A distinguishable difference between COFs and FDs was described by the first edition of the WHO classification, in which COFs presented with clear and well-defined margins in the jaws and a transition zone less than 1 mm, while FDs had poorly defined margins [29].

COFs are either uni- or multicystic lesions or mixed-density lesions [30]. The radiographic features depend on the age. Early lesions show a well-founded radiolucency accompanied by a "ground glass" radiological appearance. Time makes these lesions more radiolucent-radiopaque, with opacities appearing in the middle of the lesion with a lower density than the surrounding bone [31].

Mature lesions appear with symmetrical opacities bordered by smooth and contour peripheral osteo-condensation and an "eggshell" radiological appearance [24].

COFs presenting in the jaws can with sufficient clinical and radiographic information be diagnosed with fair certainty into one of the subcategories of fibro-osseous lesions and cannot be confirmed as COFs based on histological evidence alone [32]. Despite this fact, the finding of mature lamellar bone histologically is characteristically indicative of COFs [33,34].

The radiologic differentiation of central cemento-ossifying fibroma from Gorlin cysts and Pindborg tumours is difficult if not associated with impacted teeth, with which they have a high association; the final diagnosis is based on the histologic appearance [7,14].

The most common method in managing COFs is surgical excision. Small and well-defined lesions can be excised via enucleation and curettage, whereas large expanding lesions require radical surgery within healthy margins and aesthetic recontouring [34]. Whether to enucleate or resect radically depends on a number of factors, including involvement of the lower border of the mandible and expansion of the lesion in the adjacent soft tissues or the positioning of the maxillary sinus and nasal cavity [33,35]. Both of these surgical approaches to treating COFs have been reported to be acceptable by most authors in the literature over the past 30 years [34]. Chang et al. reported that the most common clinical sign of OF was swelling and enlargement of the buccal and/or lingual cortical plates [36]. Sciubba and Younai recommended that curettage or enucleation of the tumour should be the first line of treatment. Radiotherapy for the management of patients with ossifying fibroma is contra-indicated due to the radio resistant nature of the lesion and post-radiation complications [37]. Radiotherapy has also been shown to increase the rate of malignant transformation of the lesion from 0.4%–40% with the exclusion of certain subtypes of ossifying fibro-myxoid tumours [38]. Chemotherapeutic agents defined in the literature for the management of ossifying fibroma include the use of interferon alpha and subcutaneous calcitonin therapies. Chemotherapy for the management of aggressive juvenile ossifying fibroma in the maxilla, paranasal sinuses, or orbital COFs is beneficial, especially when using subcutaneous interferon alpha. This form of therapy has been shown to be effective in managing giant cell lesions following curettage or enucleation due to its anti-angiogenic qualities [39,40]. Merritt et al. reported a case of a juvenile ossifying fibroma of the mandible that was managed with calcitonin therapy. However, the lesion continued its progression and spread into both orbits. Therefore, the authors concluded that calcitonin therapy did not prove operational in controlling such lesions [41].

The prognosis for such lesions is considered good, while the recurrence rate is estimated to range between 0% and 28 % of cases [9,26,42]. Thorough resection is indicated in cases of recurrence [34]. Recurrence of maxillary COFs is higher than mandibular COFs due to the greater difficulty of surgical removal and their larger size at the time of presentation [2]. In the present case, the lesion in the left maxilla recurred once after surgical resection. Complete surgical removal of the lesion at the earliest possible stage has been advised by numerous investigators [43]. It is also believed that surgical intervention can reactivate the development of a lesion [43]. Therefore, an average follow-up period of 10 years after surgical treatment is vital.

## 4 Conclusion

This persistent follow-up of this rare compound case helped describe the histological, clinical, and radiological features of this lesion for a long period of time, which can help clinicians managing similar lesions to achieve good clinical, aesthetic, and functional results. Well planned, radical, and wide surgical resection of such lesions has proven not only to be effective in eliminating the aetiological factors, but can also achieve decent bone regeneration and aesthetic results with almost no deformation at the surgical site.

## Declaration of Competing Interest

The authors declare that there is no conflict of interest.

## Sources of funding

This research received no specific grant from any funding agency in the public, commercial, or not-for-profit sectors.

## Ethical approval

The ethical approval has been exempted by our institution.

## Consent

The patient received a thorough explanation of this report gave her oral and written informed consent to be included in this report as well as for publication of these case, anonymous data, and pictures. A copy of the written consent is available for review on request.

## Author’s contribution

Sarmad Aburas, Patrick Bandura and Ali Al- Ibraheem :study concept and design, writing the paper.

Marius Meier and Sebastian Berger: data collection, analysis and discussion of data.

Dritan Turhani: final approval of the version to be published.

## Registration of research studies

Not applicable. No research study involved.

## Guarantor

The corresponding author is the guarantor of submission.

## Provenance and peer review

Not commissioned, externally peer-reviewed.
